# Spatial evidence for *carcinoma* in situ (CIS) as an entity in human papillomavirus (HPV)‐associated tonsillar squamous cell carcinoma (TSCC)

**DOI:** 10.1002/ijc.70207

**Published:** 2025-10-23

**Authors:** Tobias Näsman, Madeleine Birgersson, Linda Marklund, Anders Näsman

**Affiliations:** ^1^ Department of Oncology‐Pathology Karolinska Institutet, Karolinska University Hospital Stockholm Sweden; ^2^ Department of Clinical Pathology and Cancer Diagnostics Karolinska University Laboratory (KUL), Karolinska University Hospital Stockholm Sweden; ^3^ Medical Unit Head, Neck, Lung, and Skin Cancer, Theme Cancer Karolinska University Hospital Stockholm Sweden; ^4^ Department of Clinical Sciences, Intervention and Technology, Division of ENT Diseases Karolinska Institutet Stockholm Sweden; ^5^ Department of Surgical Sciences, Section of Otolaryngology and Head and Neck Surgery Uppsala University Uppsala Sweden

**Keywords:** carcinoma in situ, dysplasia, human papillomavirus, oropharyngeal, tonsil

## Abstract

Human papillomavirus (HPV)‐associated tonsillar squamous cell carcinoma (TSCC) is suggested to arise in tonsillar crypts. It also constitutes a rare exception in the literature of carcinoma development, as carcinoma in situ (CIS) is not a recognized entity and dysplastic stages are not evident. Here we investigated the evidence of this. Hence, a systematic review and meta‐analysis was performed to study tumor origin. MEDLINE was searched and all original studies reporting tumor origin in HPV‐associated oropharyngeal cancer were included. We also used spatial transcriptomics (10× Visium) on HPV‐associated TSCC, cervical, and HPV‐independent oral cancer cases. We compared tonsillar CIS to cervical high‐grade intraepithelial lesion (HSIL). We studied epithelial cell gene expression across the sample types and epithelial cellular states. A trajectory analysis was performed in HPV‐associated tumors. In total, 14% of all TSCC were of surface origin in the systematic review. Spatial transcriptomics revealed that tonsillar CIS and HSIL clustered with a high correlation when using a pseudo‐bulk approach. Moreover, when all epithelial cells were analyzed unsupervised, ten epithelial cell clusters that correlated to histology were identified. Two of these clusters were dysplastic and occurred in all samples. A sequential progression from dysplasia to invasion was observed in both TSCC and cervical cancer in the trajectory analysis. We conclude that a subset of HPV‐associated TSCC may arise from the surface and that there is a sequential progression toward invasive disease in HPV‐associated TSCC, similar to that of cervical cancer. The findings suggest that pure tonsillar dysplastic lesions should hypothetically exist.

## Graphical abstract

What's new?

Human papillomavirus (HPV)‐associated tonsillar squamous cell carcinoma (TSCC) is suspected of originating from tonsillar crypts, where HPV can persist in latent form. Detection is further complicated by the possible absence of premalignant stages, namely carcinoma in situ (CIS). Here, the authors investigated tumor origin in HPV‐associated TSCC and performed spatial transcriptomics to characterize tumors. Meta‐analysis indicates that a subset of HPV‐associated TSCC arises from the tonsillar surface, rather than crypts. Tumors potentially also follow sequential progression toward invasive disease, similar to cervical cancer. The findings warrant further exploration to determine screening feasibility and the possible existence of pure tonsillar dysplastic lesions.

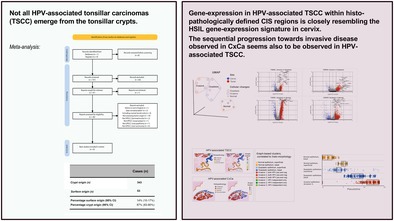

**Abbreviations**
BOTSCC: base of tongue squamous cell carcinoma
CI: confidence interval
CIS: carcinoma in situ
CxCa: cervical cancer
DEG: differentially expressed gene
FDR: false discovery rate
HGD: high‐grade dysplasia
HMSC: HPV‐related multiphenotypic sinonasal carcinoma
HPV: human papillomavirus
HSIL: high‐grade intraepithelial lesion
IHC: immunohistochemistry
ISH: in situ hybridization
OPSCC: oropharyngeal squamous cell carcinoma
OR: odds ratio
OSCC: oral squamous cell carcinoma
PC: principal component
PCR: polymerase chain reaction
SCC: squamous cell carcinoma
TSCC: tonsillar squamous cell carcinoma
UMAP: Uniform Manifold Approximation and Projection (UMAP)

## 1 INTRODUCTION

Oropharyngeal squamous cell carcinoma (OPSCC) consists of squamous cell carcinomas (SCC) of the tonsils (TSCC), the base of tongue (BOTSCC), as well as the palate and the pharyngeal walls (other OPSCC).^1^ Previous reports from others and from us have demonstrated an epidemic increase in TSCC in many Western countries, and this increase has been attributed to an increase in high‐risk human papillomavirus (HPV) infection.^2^, ^3^ Similar and parallel increases in incidence and HPV infection have also been observed for BOTSCC and OPSCC in general.^4^, ^5^, ^6^, ^7^, ^8^ Moreover, patients presenting with HPV‐associated OPSCC are generally younger and do not usually present with the classical head and neck cancer risk factors.

Despite this increase in disease burden in a young patient population, no screening methods have been established for HPV‐associated OPSCC, in contrast to HPV‐associated cervical cancer.^3^

The vast majority of all SCCs develop through a multi‐step process with sequential stages, in which normal squamous epithelium undergoes morphological dysplastic changes before eventually becoming invasive. This process is well described for most HPV‐associated SCCs, for example, cervical (CxCa), vulvar and anal SCCs, as it is for, for example, oral SCC (OSCC). Likewise, more infrequent HPV‐associated tumors, such as the newly described entity HPV‐related multiphenotypic sinonasal carcinoma (HMSC), have also been described with an association to a dysplastic epithelium. Nevertheless, HPV‐associated OPSCC is an exception to this in the literature, where high‐grade dysplasia (HGD)/carcinoma in situ (CIS) is not a recognized entity and dysplastic stages are not considered as evident.^3^, ^9^, ^10^, ^11^, ^12^, ^13^, ^14^

The cornerstone of the explanation for this biological exception has been the microenvironment of the tonsil. HPV‐associated TSCC is assumed to originate from the tonsillar crypts, which are covered by a reticulated epithelium with a discontinuous basement membrane.^15^ As a result, tonsillar neoplastic cells are suggested to have immediate access to lympho‐vascular channels, even at incipient stages, and thus considered invasive.^9^, ^10^, ^11^, ^16^

We therefore summarized the established knowledge in two sentences:

“HPV‐associated TSCC emerges from the tonsillar crypts, where the stroma‐epithelium border is indistinct, and by that HPV‐associated TSCC has immediate access to lymphovascular channels. Therefore, the histological progression through the sequential stages of surface dysplasia culminating in carcinoma in situ (CIS) and invasive growth is not evident and CIS is not an entity in HPV‐associated TSCC.”

We hypothesize that this may not be true for all HPV‐associated TSCCs. Thus, to test the first sentence, we conducted a systematic literature review and a meta‐analysis to survey if all TSCCs originate from the crypts. To test the second sentence, we utilized spatial transcriptomics on TSCC, cervical cancer (CxCa), and oral SCC (OSCC) cases harboring areas of morphologic normal epithelium, HGD/CIS, and invasive SCC. We then compared the gene expression in morphologic CIS of the tonsil to that of high‐grade intraepithelial lesion (HSIL) in the cervix and HGD in OSCC to explore possible sequential stages of TSCC progression.

## 2 MATERIALS AND METHODS

### 2.1 Systematic review and meta‐analysis: Search strategy, data extraction and statistics

A systematic review was conducted to elucidate the origin of HPV‐associated TSCC. The systematic review was registered in the PROSPERO database as “*Systematic review and meta‐analysis of tumor origin in HPV‐related oropharyngeal squamous cell carcinomas*,” with the ID: CRD42024548127. The PRISMA 2020 statement was consulted to perform the systematic review.^17^ Using PubMed, the MEDLINE database was searched for all types of original studies (excluding reviews) in English published until June 2024. The search terms were (HPV OR Papillomaviridae[MeSH]) AND (oropharyngeal OR oropharynx OR tonsil OR tonsillar OR “base of tongue” OR “soft palate” OR oropharyngeal* OR tonsil*) AND (crypt OR crypt* OR reticulated OR reticul* OR surface OR surfac*) NOT (Review[Publication Type]). The search was performed on June 15, 2024.

Two researchers (TN and AN) reviewed all generated abstracts individually and independently. Abstracts containing any description or term/word of crypt, surface, reticulated or dysplasia were selected for full‐text review. Articles selected by either researcher were included.

**FIGURE 1:**
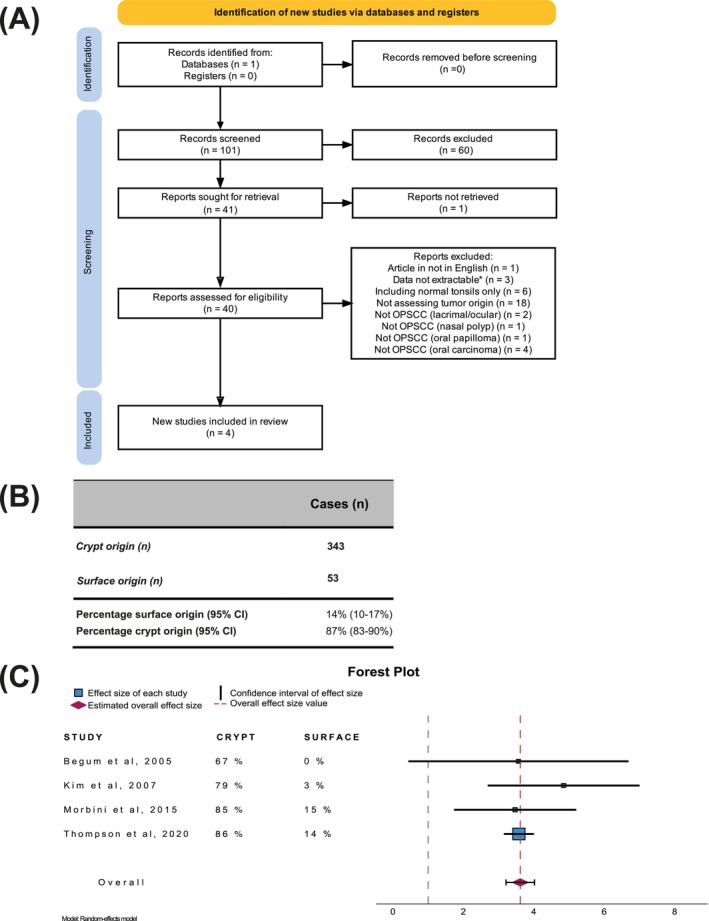
Data from meta‐analysis and systematic review. (A) Flow diagram of the study population identification and selection process. For the detailed and entire stepwise selection process, please see Supplemental Table 1. In total four articles were selected for data analysis. (B) Table showing cryptal and surface origin in oropharyngeal squamous cell carcinoma. 14% (95% CI: 10–17) were of surface origin. (C) Forest plot with odds ratios (OR) of having an HPV‐associated oropharyngeal cancer with cryptal origin. The percentages of cryptal and surface origin are stated per study. The overall OR for having a cryptal origin was 3.6 (95% CI: 3.2–4.0).

The selected articles were reviewed by TN and AN. Data on tumor origin, presence, and localization of dysplasia and tumor center were extracted by both reviewers independently. Additionally, the diagnosis of HPV‐associated TSCC was confirmed in each article (regardless of diagnostic criteria, i.e., p16, ISH, PCR). In case of disagreement, the data presented in the article were discussed, and a consensus was reached. If data were presented but not extractable, the corresponding author was contacted and was given 2 weeks to respond. If no response was received within that time, or if the data were unavailable, the article was excluded. The entire stepwise selection process, including data extraction, is summarized in a flow‐chart^18^ (Figure 1) and in an Excel spreadsheet (Supplemental Table 1).

### 2.2 Patients and tumor material

To enable analysis of gene expression using spatial transcriptomics, the Karolinska Hospital registry was queried for patients with HPV‐associated TSCC, as defined by clinical practice (p16 positive by immunohistochemistry using the CINtec® p16 antibody *and* HPV DNA positive by the BD Onclarity™ HPV Assay) during 2023. In total, 44 patient cases were identified, and their pre‐treatment specimens were reviewed by histology. Cases harboring normal epithelium, epithelium showing characteristics of HGD/CIS, and invasive carcinoma within the same tissue slide were sought. Three such cases were identified and selected. Importantly, all three cases contained these three cellular states within an area of 6 mm^2^, matching the Visium 10× array capture area. Thereafter, three consecutive cases of HPV‐independent oral squamous cell carcinomas (OSCC) from 2023 were selected from the same registry, and two anonymized HPV‐associated cervical squamous cell carcinoma (CxCa) cases were also obtained for controls. All controls contained normal epithelium, HGD/HSIL, and invasive carcinoma within an area of 6 mm^2^ in the same tissue slide. However, one anonymized CxCa case first had to be cut and re‐embedded to have such representation.

Patient data from the HPV‐associated TSCC cases collected from the patients' clinical records (Supplemental Table 2).

### 2.3 Histological annotations of tissue slides

**FIGURE 2:**
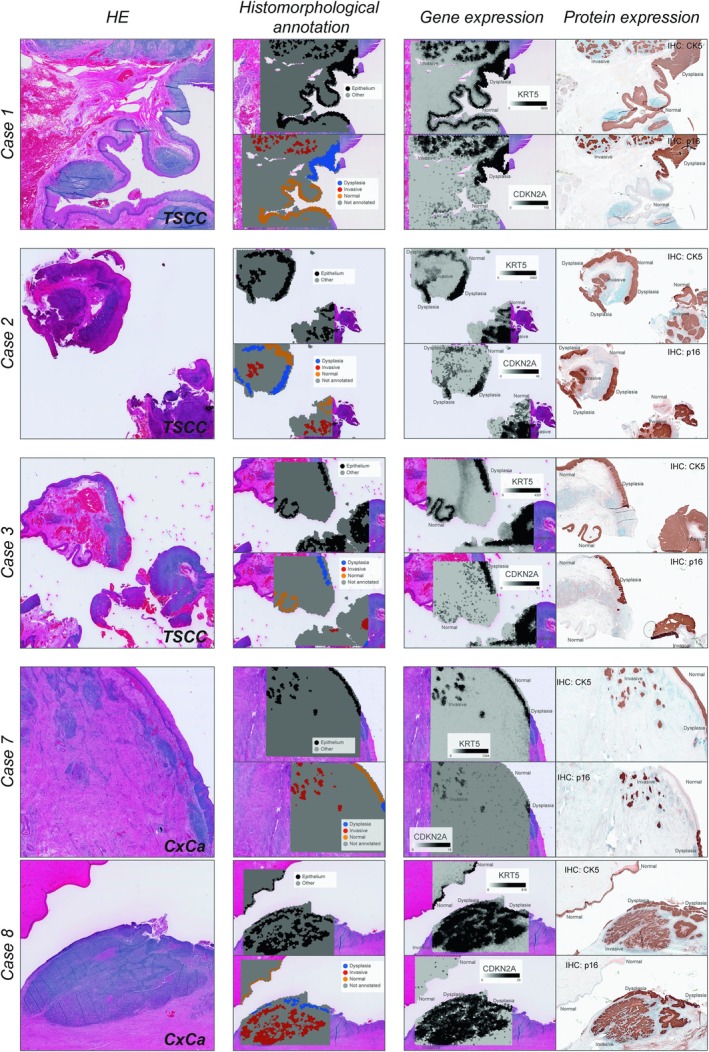
The five included cases with HPV‐associated tonsillar squamous cell carcinoma (TSCC) and cervical squamous cell carcinoma (CxCa), presented by their histomorphology (HE), their manual annotations (Histomorphological annotation), their spatial gene expression (KRT5 and CDKN2A) and their protein expression (CK5 and p16) by immunohistochemistry. The protein expression of CK5 was visually highly correlated with the gene expression of KRT5 and histomorphological annotations (epithelium). Likewise, p16 protein expression was visually highly correlated with CDK2NA gene expression and morphological annotations (dysplasia and invasive). We therefore considered the gene‐expression of the Visium assay, and our annotations validated against the two clinically important IHC protein markers.

Slides were annotated for further analysis by spatial transcriptomics. All eight tumor slides were annotated for histomorphology (“Epithelium” and “Other”) and for histopathology (“Normal”, “Dysplasia” and “Invasive”) by two surgical pathologists (TN and AN), for use in spatial transcriptomic analysis. The 10× Visium assay's capture area contains 4992 barcoded spots. Thus, the method entails that annotations are made in spots, and for every spot a consensus was made. Spots annotated as “epithelium” were assessed to contain an area with >70% squamous epithelial cells. Likewise, spots annotated for histopathology were assessed to contain >70% of the annotated cellular state. In addition, only spots in which there was no diagnostic ambiguity by any of the researchers regarding their histopathological state were annotated. As a result, fewer spots were annotated for histopathology than for histomorphology (Figure 2 and Supplemental Figure 1). All annotations were performed in the 10× Loupe browser (v 6.5.0, 10× Genomics, Leiden, The Netherlands).

### 2.4 Sample preparation, library preparation and RNA sequencing

Validated protocols by 10× Genomics were used for the sample and library preparation. For tissue preparation, the Visium CytAssist Spatial Gene and Protein Expression—FFPE Tissue Preparation Guide (Document CG000660) was followed. The document Visium CytAssist Spatial Gene and Protein Expression—Deparaffinization, H&E Staining, Imaging & Decrosslinking for FFPE Tissue (Document CG000658) was followed in the tissue staining, and the document Visium CytAssist Spatial Applications Imaging Guidelines (Document CG000521) was followed in the imaging. For library construction and sequencing, the Visium CytAssist Spatial Gene and Protein Expression Reagent Kits (Document CG000494) were followed. The sequencing statistics for each sample are summarized in Supplemental files 1–8.

### 2.5 RNA‐seq data processing and clustering

All analyses stated below were performed in the software Partek Flow (v. 12.2.0, Partek, San Diego, CA) using default settings unless otherwise stated. All reads were initially filtered on histomorphology; therefore, including counts from morphological squamous epithelial cells only. Thereafter, we filtered out low‐quality cells, in this instance defined as those with fewer than 200 counts and ribosomal counts of <2. Subsequently, normalization (CPM) and sample batch removal (Seurat3 integration) were performed. After this, data were explored by principal components analysis using the top 2000 features with the highest variance. The top 20 significant principal components (PCs) were selected for Uniform Manifold Approximation and Projection (UMAP) dimension reduction and visualization of gene expression. A graph‐based clustering (Louvain clustering algorithm with number of nearest neighbors = 30 and number of random starts = 100) was performed.

Normalized counts were filtered on histopathologically defined epithelial cells, consequently including cells annotated as “*Normal*,” “*Dysplasia*,” or “*Invasive*” in HPV‐associated tumors (CxCa and TSCC) only. A pseudo‐bulk approach was used, where histomorphological annotations were bulked per sample and site by using mean values. Data were then visualized in a UMAP (top 10 PC) or as in a similarity matrix (Correlation method: Pearson).

Differentially expressed genes (DEGs) per tumor site in HPV‐associated tumors were determined by Partek Flow's gene differential filter analysis using ANOVA. Genes with a false discovery rate (FDR) <0.05 were filtered and a gene‐set enrichment (using KEGG database) was performed.

### 2.6 Trajectory analysis

To explore stages of cancer development, we performed a trajectory analysis on all the epithelial cells in HPV‐associated tumors using Monocle3, integrated into the Partek Flow software (v. 12.2.0, Partek, San Diego, CA). We used epithelial cells annotated as “Normal” as the programmatically calculated default root nodes.

### 2.7 Immunohistochemistry

Immunohistochemistry (IHC) was used to correlate protein expression with gene expression from spatial transcriptomics and with epithelial and dysplastic annotations. All IHC staining was performed on the Ventana BenchMark Ultra platform, using the ready‐to‐use antibodies p16 (CINtec®, Roche) and CK5 (clone SP27, Roche). Heat Induced Epitope Retrieval (HIER) was performed with CC1 solution for 48 and 64 min for p16 and CK5, respectively. Incubation with primary antibodies was performed in 24 and 32 min for p16 and CK5, respectively.

### 2.8 Statistical analysis

All statistical analyses of the extracted data were performed with IBM Corp., SPSS Statistics, version 29.0. The meta‐analysis option for binary outcomes was used to calculate pooled odds ratios (OR) with 95% confidence intervals (CI) across studies using the random‐effects model and to visualize data in a forest plot. Heterogeneity was evaluated using the Cochran's *Q* test, the *I* ^2^ statistics, and the Tau^2^.

## 3 RESULTS

### 3.1 Do all TSCC emerge from tonsillar crypts? A systematic review and meta‐analysis

To evaluate the current evidence supporting the proposition that *TSCCs arise from the tonsillar crypts*, we performed a systematic review and meta‐analysis. We identified 101 articles, of which 40 had an abstract stating any of the terms “*crypt*”, “*reticulated*”, “*surface*” or “*dysplasia*” in the abstract text and were retrievable. Among these 40 manuscripts, seven articles addressed tumor origin, all of which favored a cryptal origin. However, data could only be extracted from four of these seven articles (Figure 1A, Supplemental Table 1).^19^, ^20^, ^21^, ^22^

The resulting four studies were all retrospective cohort studies, and while some examined tumor origin as a primary study aim,^19^, ^21^, ^22^ others reported origin as a secondary finding.^20^ Nevertheless, when summarizing the data, 87% (95% CI: 83%–90%) of the tumors were deemed as having a cryptal origin (Figure 1B), whereas 14% (95% CI: 10%–17%) were deemed as having a surface origin. The overall OR for a cryptal origin was 3.6 (95% CI: 3.2–4.0, *p* <.001) (Figure 1C). The heterogeneity was low (*I* ^2^ = 4.7%), the *Q*‐test was not significant (*Q* = 4.2, *p* = .38), and the Tau^2^ was 0.01, suggesting a high level of consistency among the included studies. Taken altogether, the compiled published data support the notion that the majority of TSCC cases originate in the tonsillar crypts, but notably, more than 10% of all TSCC originate at the tonsillar surface (Figure 1B).

### 3.2 Patient cases harboring normal, HGD/CIS and invasive epithelial cells

To examine if CIS in tonsillar tissue differs from invasive or normal tonsillar epithelium, and whether there are similarities between CIS in tonsils and HSIL in the cervix, we searched for recent TSCC cases in our hospital pathology register.

In total, three HPV‐associated TSCC cases were identified, each containing normal epithelium, epithelium with morphological findings consistent with HGD/CIS, and invasive SCC in the same tissue section (Figure 2). The patients' clinical characteristics are depicted in Supplemental Table 2. Briefly, two of the patients were diagnosed by tonsillectomy, and one patient was diagnosed by surgical biopsy. All tumors demonstrated overexpression of p16 by immunohistochemistry (>70%), and two cases harbored HPV‐16 DNA, while the third harbored HPV‐18 DNA. All three tumors were classified as stage I tumors according to TNM‐8. All three patients showed complete response to treatment and were disease‐free and alive at the time of last check‐up (Supplemental Table 2).

Thereafter, three cases with p16‐negative OSCC (originating from the mobile tongue) and two anonymized p16‐positive CxCa cases were collected. Subsequently, all eight tumors were analyzed with the 10x Visium assay and annotated by their histomorphology and histopathology in the Loupe Browser (see Material and Methods). Importantly, we validated the gene expression of the Visium assay against clinically important IHC protein markers used in routine clinical practice. There was a high agreement between the gene expression of KRT5 and CDKN2A and the protein expression of CK5 and p16 respectively (Figure 2 and Supplemental Figure 1). Protein expression of CK5 is often used to visualize squamous epithelial cells, and the correlation with gene expression of KRT5 as well as our epithelium annotations correlated well. Likewise, p16 protein expression is often used as a surrogate marker for high‐risk HPV, and the correlation between p16 and CDKN2A as well as the correlation between CDKN2A and high‐grade dysplasia/invasive carcinoma in HPV‐associated cases was also high.

### 3.3 CIS in oropharynx is similar to HSIL in cervix

**FIGURE 3:**
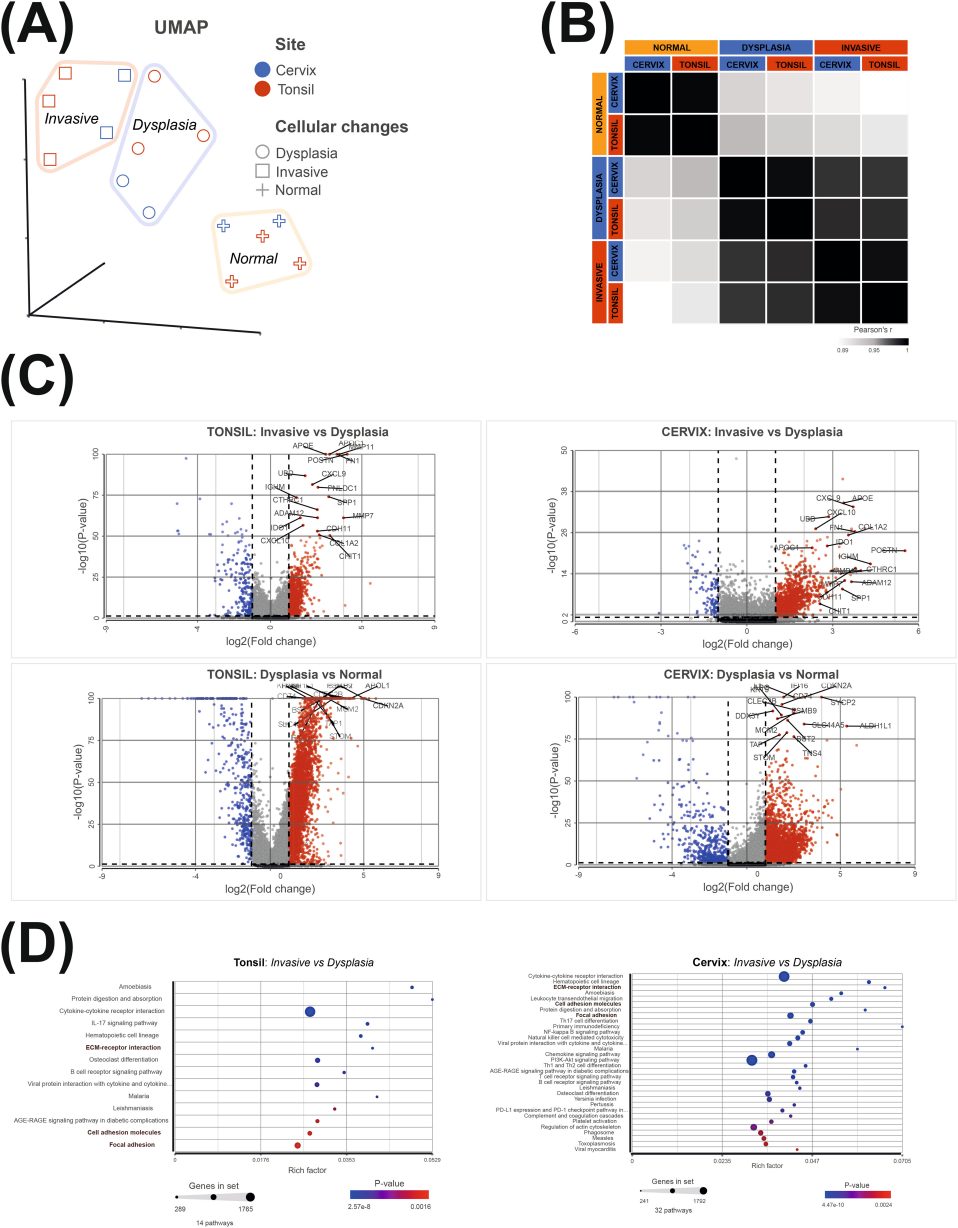
Comparisons of tonsillar and cervical normal, dysplastic and invasive epithelium, using a pseudo‐bulk approach. (A) UMAP with pseudo‐bulks per histopathological annotations and patient case. (B) Similarity matrix with pseudo‐bulks per histological annotation and anatomical site, showing highest correlation within histological annotations. (C) Volcano plots with differentially expressed gene (DEG) analysis. The top significantly DEGs between invasive and dysplasia in tonsils, were also all significantly differentially expressed in cervix. Similarly, the top significantly DEGs between dysplasia and normal in cervix, were all significantly expressed in the tonsil. (D) Gene set enrichment analysis including the top DEGs (genes (FDR <0.05 and fold change ± 2)) between invasive and dysplasia per anatomical site. The KEGG‐pathways “ECM‐receptor interaction,” “Cell adhesion molecules,” and “Focal adhesion” were all three enriched independent of site.

We started the analysis by examining similarities between histopathologically defined HGD/CIS in tonsils and HSIL in the cervix. We used a pseudo‐bulk approach, where all epithelial cells with identical histopathological annotation (Normal, Dysplasia, or Invasive) were merged per individual case in the HPV‐associated tumors. Epithelial cells with ambiguous histology were excluded (see Material and Methods). In a UMAP, we observed a separation between “Normal,” “Dysplasia” (high‐grade), and “Invasive” pseudo‐bulks, where these cellular states clustered independent of anatomical origin (Figure 3A). Importantly, the high‐grade tonsillar pseudo‐bulks clustered with the corresponding HSIL pseudo‐bulks, rather than with the tonsillar invasive pseudo‐bulks (Figure 3A). Thereafter, we performed another pseudo‐bulk, but now per site of origin instead of per case. Again, the histopathologically defined cellular states (Normal vs. Dysplasia vs. Invasive) correlated, rather than site of origin (Figure 3B). Notably, HGD/CIS in tonsils had the highest correlation with HSIL in the cervix.

Then, a DEG analysis was performed, where genes differently expressed between invasive versus dysplasia and dysplasia versus normal were assessed per site in HPV‐associated tumors (Figure 3C). Importantly, the top‐overexpressed genes between “Invasive” versus “Dysplasia” in tonsils (cut‐off: log2 fold change = 2 and *p*‐value = −log10[50]) were all found significantly overexpressed in cervical “Invasive” versus “Dysplasia” (Figure 3C). Similarly, the top overexpressed genes in cervix between “Dysplasia” versus “Normal” (cut‐off_ log2 fold change = 2 and *p*‐value = −log10(75)) were also all significantly differentially expressed between “Dysplasia” vs. “Normal” in tonsils (Figure 3C).

Lastly, when assessing the top differentially expressed genes (FDR <0.05 and fold change ± 2) between “Invasive” vs. “Dysplasia” in a gene set enrichment analysis, “ECM‐receptor interaction,” “Cell adhesion molecules,” and “Focal adhesion” were all three enriched KEGG‐pathways, independent of site (Figure 3D).

### 3.4 “Unsupervised” cluster analysis identifies similar clusters in HPV‐associated TSCC and CxCa and the clusters correlate to histomorphology

**FIGURE 4:**
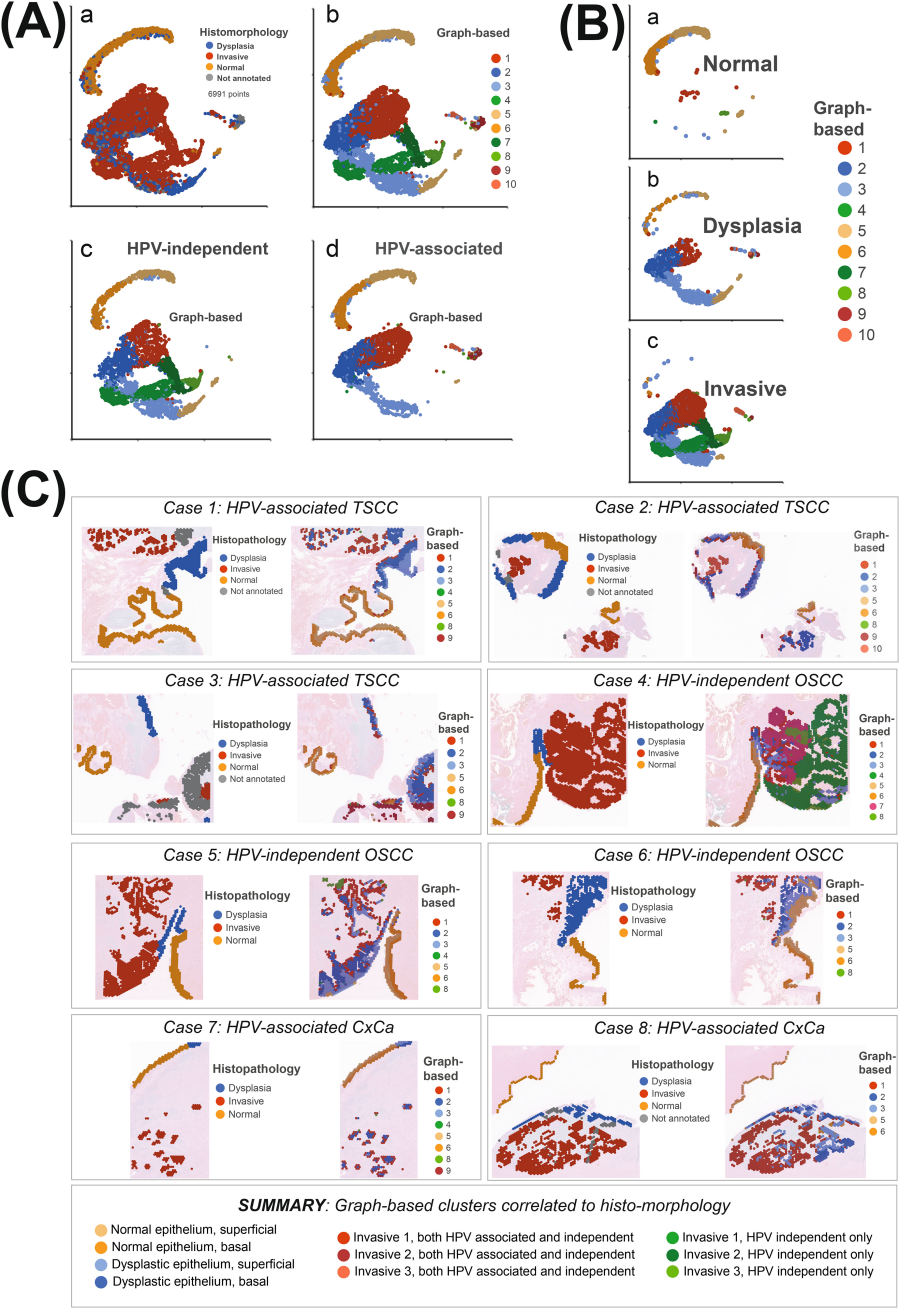
Ten epithelial cell clusters, identified after graph‐based clustering (Louvain). (A) (a) UMAP with all 6991 cellular spots, annotated by their histological states (Histomorphology). Some spots not annotated due to diagnostic ambiguity. (b) UMAP with all 6991 cellular spots, annotated by the ten graph‐based clusters. (c), (d) UMAPs with the ten graph‐based clusters separated on HPV status. Three clusters (4, 7, and 8) were unique to HPV‐independent tumors (OSCC). (B) UMAPs presenting the ten graph‐based clusters separated on the histopathological annotations. Clusters 5–6 were overrepresented in the “normal” epithelium, while clusters 2–3 in the dysplastic epithelium. The others were overrepresented in the invasive epithelium. (C) The ten graph‐based clusters annotated in their spatial context with manual histomorphological annotation for comparison. The graph‐based clusters correlated with histomorphology and changed the histomorphological interpretation in case 8.

We then performed an “unsupervised” analysis on all epithelial cells, independent of HPV status, tumor site, and histopathological annotation, to examine similarities in clusters between TSCC, CxCa, and OSCC. For this purpose, we included all epithelial cells (6991 spots in all eight samples) and performed a graph‐based clustering (Louvain), which resulted in ten epithelial clusters. Figure 4A depicts all 6991 cellular spots, which are annotated with histopathological cellular states in a UMAP (a) and by the ten graph‐based clusters (b). When we separated the epithelial cells based on HPV status (Figure 4A, c‐d), three clusters seemed unique for HPV negative controls, while the other seven were all present in all cases independent of HPV status.

Moreover, when we separated the clusters on the histopathological annotations in three UMAPs (Figure 4B), we observed that clusters 5–6 were overrepresented in the “normal” epithelium and clusters 2–3 (and to some extent also cluster 1) in the dysplastic epithelium, while the other clusters were overrepresented in the invasive epithelium.

To further examine the agreement between the graph‐based clusters and histomorphology, all epithelial cells, previously also annotated for histopathological cellular states, were now also annotated for the graph‐based clusters (Figure 4C). When summarizing the correlation between histology and the graph‐based clusters, we found that clusters 6 and 5 corresponded to normal basal and superficial epithelium, respectively, while clusters 2 and 3 corresponded to dysplastic basal and superficial epithelium, respectively. The other clusters all corresponded to invasive epithelium (Figure 4C).

Furthermore, when we scrutinized the histopathology again, with the graph‐based cluster annotations available (Figure 4C), it became evident to us that some cell spots had been misinterpreted in the initial annotation. As an example, in case 8 (CxCa) a small area was annotated as invasive but was part of a dysplastic cluster according to graph‐based clustering. Accordingly, after a second review, there was an agreement (between TN and AN) that this area rather represented HSIL (i.e., dysplasia per annotation) and not invasive epithelium.

Interestingly, in most cases, and especially pronounced in the three HPV‐independent cases, there was a transition from a dysplastic epithelium into a hyperplastic dysplastic epithelium, in which the basal cells belonged to the “Invasive 1” cluster, and the more superficial cells to dysplastic clusters. These findings support the widely accepted notion that malignant transformation is initiated in the basal layer of the epithelium in SCC.

### 3.5 Trajectory analysis

To further explore if HPV‐associated TSCC is preceded by dysplasia, we built trajectories for HPV‐associated TSCC and CxCa using Monocle 3. Based on epithelial maturation, squamous cell carcinoma development and data obtained above (Figure 4), the value for root nodes was set to “Normal” epithelium. The trajectory was thereafter annotated by histomorphology (A) and by the graph‐based clusters (B). We identified two different chronological directions of the trajectory. Starting in normal basal epithelium, the trajectory could either shift toward cell maturation or proceed in malignant transformation. Notably, when assessing pseudotime in the cluster‐based annotated cells, it became evident that the basal normal epithelium preceded superficial normal epithelium, while superficial dysplastic epithelium preceded basal dysplastic epithelium. This finding suggests that the core events in epithelial transformation, or maturation, take place in the basal epithelial cells.

**FIGURE 5:**
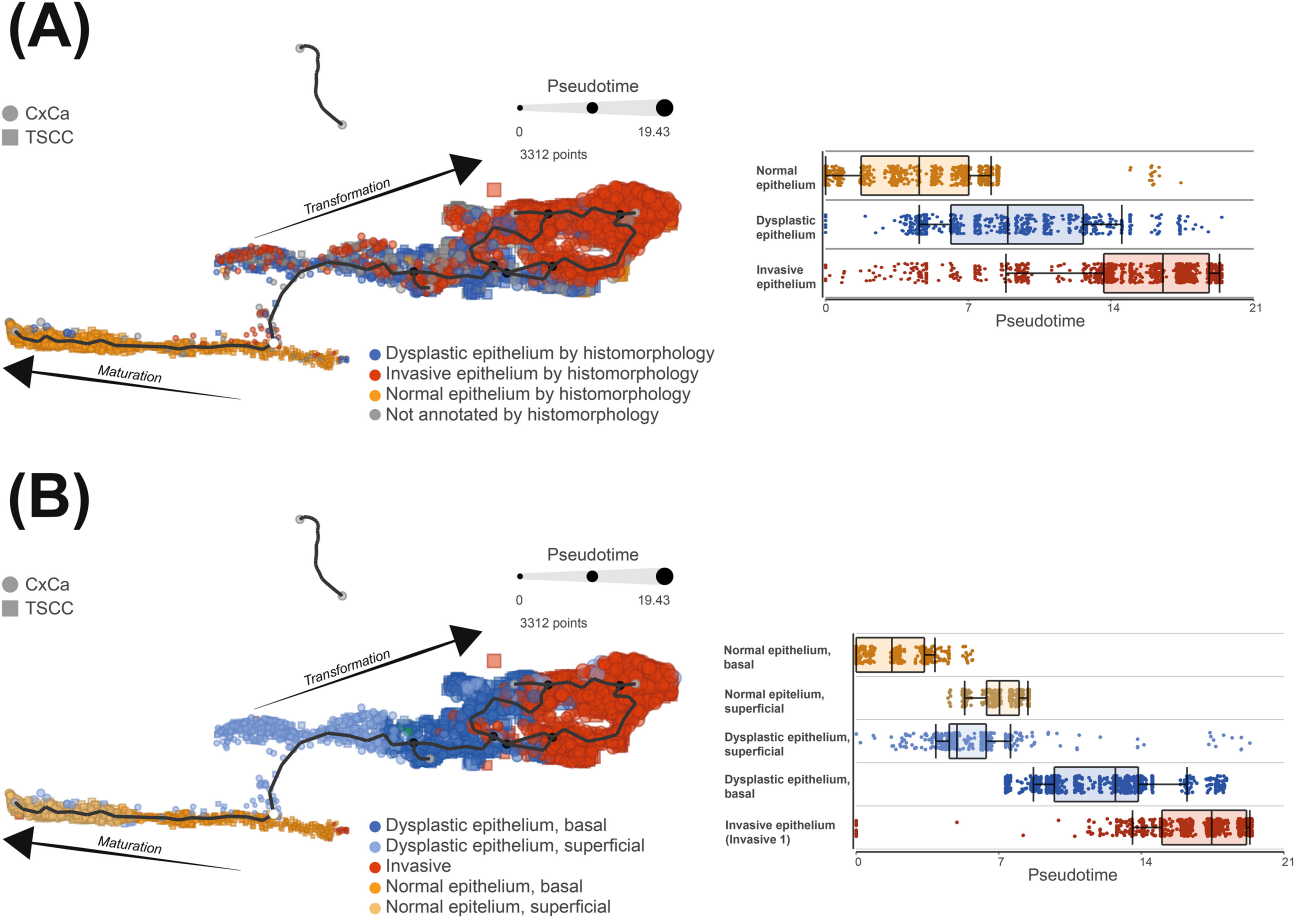
Trajectory analysis of HPV‐associated tumors, annotated by histomorphology (A) and by the ten graph‐based clusters, named by the results from Figure 4B. Starting in “Normal basal epithelium” (white dot), the trajectory bifurcates into “Maturation” or into “Transformation.” The different pseudotimes are presented as box plots. Epithelial cellular spots from both cervix and tonsil are evenly spread and admixed along the whole trajectory.

Nevertheless, regardless of maturation or transformation, there was a mixture of cervical and tonsillar epithelial cells along the whole trajectory, and this mixture was present at all annotated stages (Figure 5A,B). Taken together, these results suggest that there is a sequential progression from normal epithelium into dysplasia, followed by invasion, and that this sequential progression is present in both CxCa and TSCC.

## 4 DISCUSSION

In this study, we identified a gene‐expression signature in HPV‐associated TSCC within histopathologically defined CIS regions, closely resembling the HSIL gene‐expression signature in CxCa. We also demonstrated that the sequential progression toward invasive disease observed in CxCa is also observed in HPV‐associated TSCC. Lastly, we determined that more than 10% of all HPV‐associated TSCCs arise from the tonsillar surface, suggesting that a proportion of TSCCs may not arise in the crypts. We therefore suggest that CIS in TSCC should be regarded as an entity, similar to that of HSIL in the cervix. From a broader perspective, these results suggest that pure HGD/CIS lesions of the tonsil may hypothetically exist.

The multi‐step sequential carcinoma development model is now well established and applies to the majority of all carcinomas. Likewise, it is well established that HPV‐associated CxCa develops from HSIL, as a result of high expression levels of the HPV viral oncogenes.^12^ Similarly, premalignant stages of all other HPV‐associated anogenital carcinomas are also well established. However, HPV‐associated premalignant lesions are not unique to the anogenital area. They have also been described in, for example, the head and neck region. As also stated above, the newly described entity HMSC has been associated with HGD and HPV‐associated oral epithelial dysplasia is a recognized premalignant entity of the oral cavity by WHO.^11^, ^14^, ^23^, ^24^ In addition, although rare, early studies in the field have reported the presence of dysplasia in HPV‐associated TSCC. In a study by Begum et al., dysplasia was present in 8 out of 8 tonsils adjacent to the invasive carcinoma. Interestingly, dysplasia was also present in 1 out of 8 contralateral tonsils.^22^ Notably, similar findings are rarely reported today, likely due to updated WHO classifications and evolving histopathological criteria. Here, we show that HSIL and tonsillar HGD/CIS both correlate to each other and that they both separate from invasive and normal epithelium—suggesting that tonsillar CIS should be recognized as an entity, just as HSIL is in the cervix.

We also demonstrated a sequential progression into invasive disease, both in TSCC and in CxCa, which appears reasonable from a biological standpoint. Nevertheless, no pure tonsillar CIS lesion has been described, although some attempts to identify such have been performed.^3^ The largest such study, to our knowledge, was published in 2014 and included 4095 patients.^25^ In that study, the authors found no evidence of HPV‐associated premalignant neoplasia, since all tonsils were negative for HPV DNA by PCR. However, it is worth noting that only around 10% of the study population were older than 45 years old, placing them outside the primary risk group for HPV‐associated TSCC. Besides, risk factors for having a tonsillar HPV infection and having an indication for an elective tonsillectomy may not be overlapping. In addition, as a more general challenge in these kinds of studies, the sensitivity of PCR‐based HPV DNA detection methods may be reduced due to too much human DNA included in the PCR‐assay, which reduces the PCR's ability to detect viral DNA. There might also be sampling errors in these kinds of studies when only a section from the tonsillar epithelium is included in the assay.^26^ Therefore, larger studies, including older patients with stratification for risk factors and multiple samples from the same tonsil, are probably needed to detect such pure premalignant lesions.

Nonetheless, previous studies have demonstrated that HPV16 E6 seropositivity is present >10 years before diagnosis of OPSCC,^27^ suggesting that a chronic persistent epithelial HPV infection precedes invasive disease. Notably, persistent cervical HPV infection beyond 12 months is an established risk factor for HSIL and potentially invasive carcinoma.^28^, ^29^ Our results point to the possibility that persistent HPV infection may induce premalignant dysplastic changes in tonsillar epithelial cells, similar to those in the cervical epithelium.

Notably, our meta‐analysis demonstrated that a significant proportion of HPV‐associated TSCCs arise in the crypts. The low heterogeneity supports the robustness of the pooled OR, and the similarities in effect sizes across the studies strengthen the today accepted notion that cryptal involvement is common in these tumors. However, different or even unclear histopathological criteria for defining cryptal origin, as well as sampling issues, may influence the generalizability of the findings. Nevertheless, a subset of HPV‐associated TSCCs still appears to originate from the surface. These findings are in line with the established knowledge of cervical cancer development, stating at least three progression routes dependent on the initially infected epithelial cell type. Two of these cervical routes of progression have parallels in the tonsils. Most cervical carcinomas arise in the transformation zone (*corresponding to tonsillar crypts*) and are suggested to be associated with a higher risk of cancer progression. However, they may also arise in the ectocervix (*corresponding to tonsillar surface*), where the progression from dysplasia to invasive disease is slow.^29^ When translating these findings into TSCC, it is possible that the dominance of cryptal origin leads to fast progression into invasive disease, partly explaining why pure premalignant lesions have not been identified.

The existence of pure premalignant lesions in tonsils may in a broader perspective allow for screening.^13^, ^30^, ^31^ The results presented here suggest screening should be theoretically possible, that is, presence of early lesions that can be surgically treated. However, the question remains whether screening is feasible and which screening method to employ.^32^ As an example of difficulties with screening, attempts have been made with mouthwashes to detect HPV DNA. The HPV prevalence has been reported to vary in healthy populations and with lower HPV DNA quantities, as compared to cervical samples, suggesting that HPV infection is more difficult to detect in oral than cervical samples.^3^, ^33^ In line with that, oral HPV DNA was in a study only reported in 50% and 82% in patients with diagnosed HPV‐associated BOTSCC and TSCC, respectively.^30^ Notably, detection of oral HPV DNA was in that study also associated with tumor size and especially noteworthy 0% of patients with stage T1 HPV‐associated tumors had detectable HPV DNA in their saliva.^30^ Furthermore, in another study, prevalence of HPV was determined in mouthwashes and in tonsils in patients undergoing tonsillectomy.^26^ No association between presence of oral HPV and presence of HPV in tonsils was reported in that study and, as discussed above, HPV DNA has rarely been detected in healthy tonsils.^25^, ^34^ Taken together, even if screening for premalignant tonsillar lesions is theoretically possible, it may not necessarily be feasible.

We acknowledge that a limited number of cases were included (*n* = 8), which is a key limitation in this study. Indeed, there is a high variability between patients and their samples, and most likely also within each individual tumor sample. Consequently, sampled regions may not fully reflect the total tumor biology and some spatial expressions may rather represent local microenvironmental features than global tumor characteristics. Therefore, these data should be interpreted with some caution, particularly with regard to the expression of individual genes across the epithelial stages. Nonetheless, despite the fact that only five HPV‐associated carcinomas were studied, we were able to demonstrate global similarities between these tumor types, suggesting the presence of pre‐malignant lesions in the tonsil.

In summary, we show that cryptal origin is not obligatory in all HPV‐associated TSCCs and that these tumors may follow a sequential progression toward invasive disease. The findings suggest that CIS in TSCC is an entity and that pure tonsillar dysplastic lesions should hypothetically exist. From a broader perspective, these findings suggest that screening for HPV‐associated TSCC could be possible; however, whether that is achievable or not remains to be elucidated.

## AUTHOR CONTRIBUTIONS

**Tobias Näsman:** Conceptualization; investigation; writing – original draft; writing – review and editing; data curation; visualization; methodology; formal analysis; project administration; software; validation. **Madeleine Birgersson:** Investigation; writing – review and editing; data curation; formal analysis; validation. **Linda Marklund:** Investigation; writing – review and editing; data curation; formal analysis; validation. **Anders Näsman:** Conceptualization; investigation; funding acquisition; writing – original draft; writing – review and editing; data curation; supervision; visualization; methodology; formal analysis; project administration; resources; software; validation.

## FUNDING INFORMATION

This work was funded by The Swedish Cancer Society, Radiumhemets forskningsfonder, Cancer och Allergifonden, Åke Wibergs stiftelse and Karolinska institutet. The funding bodies had no role in the study design, data collection, analysis, interpretation of data, or in writing the manuscript.

## CONFLICT OF INTEREST STATEMENT

The authors have declared that no conflict of interest exists.

## ETHICS STATEMENT

Consent was obtained, when appropriate, from subjects involved in the study according to the ethical permission 2023–01911‐01 (Ethical Committee in Stockholm, Sweden). The study was approved by the Ethical Committee in Stockholm, Sweden, according to the ethical permission 2023–01911‐01.

## Supporting information


**Supplemental Table 1.** Detailed selection process of articles in the meta‐analysis and systematic review.

**Supplemental Table 2.** Patients with HPV‐associated tonsillar squamous cell carcinoma (TSCC) and their characteristic.
**Figure S1.** The three included cases with HPV‐independent oral squamous cell carcinoma (OSCC), presented by their histomorphology (HE), their manually annotations (histomorphological annotation), their spatial gene expression (KRT5 and CDKN2A) and their protein expression (CK5 and p16) by immunohistochemistry. The protein expression of CK5 was highly correlated visually to gene expression of KRT5 and histomorphological annotations (epithelium). No overexpression of p16 protein by IHC, nor any CDK2NA gene overexpression was observed. Therefore, we considered the gene‐expression of the Visium assay, and our annotations validated.

## Data Availability

Data that support the findings of this study may be made available from the corresponding author upon reasonable request, after legal requirements have been met and appropriate permissions have been obtained. The RNA seq data generated in this study are available under restricted access in the Swedish National Data Service (SND) portal at www.researchdata.se, under the identifier (DOI): https://doi.org/10.48723/v39q-2169.
