# Supplementary material for: Spatial evidence for carcinoma in situ (CIS) as an entity in human papillomavirus (HPV)‐associated tonsillar squamous cell carcinoma (TSCC)
Source: Int J Cancer. 2025 Oct 23;158(4):1080–92. doi: 10.1002/ijc.70207 (PMC12712372; doi:10.1002/ijc.70207)
Supplement: Supplementary file 2 — Supplemental Table 2. Patients with HPV‐associated tonsillar squamous cell carcinoma (TSCC) and their characteristic. Figure S1. The three included cases with HPV‐independent oral squamous cell carcinoma (OSCC), presented by their histomorphology (HE), their manually annotations (histomorphological annotation), their spatial gene expression (KRT5 and CDKN2A) and their protein expression (CK5 and p16) by immunohistochemistry. The protein expression of CK5 was highly correlated visually to gene expression of KRT5 and histomorphological annotations (epithelium). No overexpression of p16 protein by IHC, nor any CDK2NA gene overexpression was observed. Therefore, we considered the gene‐expression of the Visium assay, and our annotations validated. [file IJC-158-1080-s001.pdf]

Spatial evidence for *carcinoma in situ (CIS)* as an entity in human papillomavirus (HPV)-associated tonsillar squamous cell carcinoma (TSCC).

Tobias Näsman, Madeleine Birgersson, Linda Marklund, Anders Näsman

Table of content

**Type of content**	**Page below, or as separate file/s**
1. **Supplemental Table 1.** Detailed selection process of articles in the meta-analysis and systematic review.	Separate Excel file (Supplemental Table 1)
**2. Supplemental Table 2** Patients with HPV-associated tonsillar squamous cell carcinoma (TSCC) and their characteristic.	Page 2
**3. Supplemental Figure 1 (Figure S1).**	Page 3
**4. Supplemental Files 1-8** Space ranger counts per case (case 1-8).	Page 4-11

Supplemental Table 2. Patients with HPV-associated tonsillar squamous cell carcinoma (TSCC) and their characteristic.

Case	HPV DNA	p16	TNM-8 Stage	T	N	M	Treatment	Response to treatment	Alive at last check-up	Tumor-free	Follow-up time (days)
Case 1	HPV 16	Positive	I	1	1	0	CRT (68Gy and Cisplatin weekly)	CR	Yes	Yes	637
Case 2	HPV 18	Positive	I	2	1	0	RT (68 GY)	CR	Yes	Yes	342
Case 3	HPV 16	Positive	I	2	1	0	RT (68 GY)	CR	Yes	Yes	331

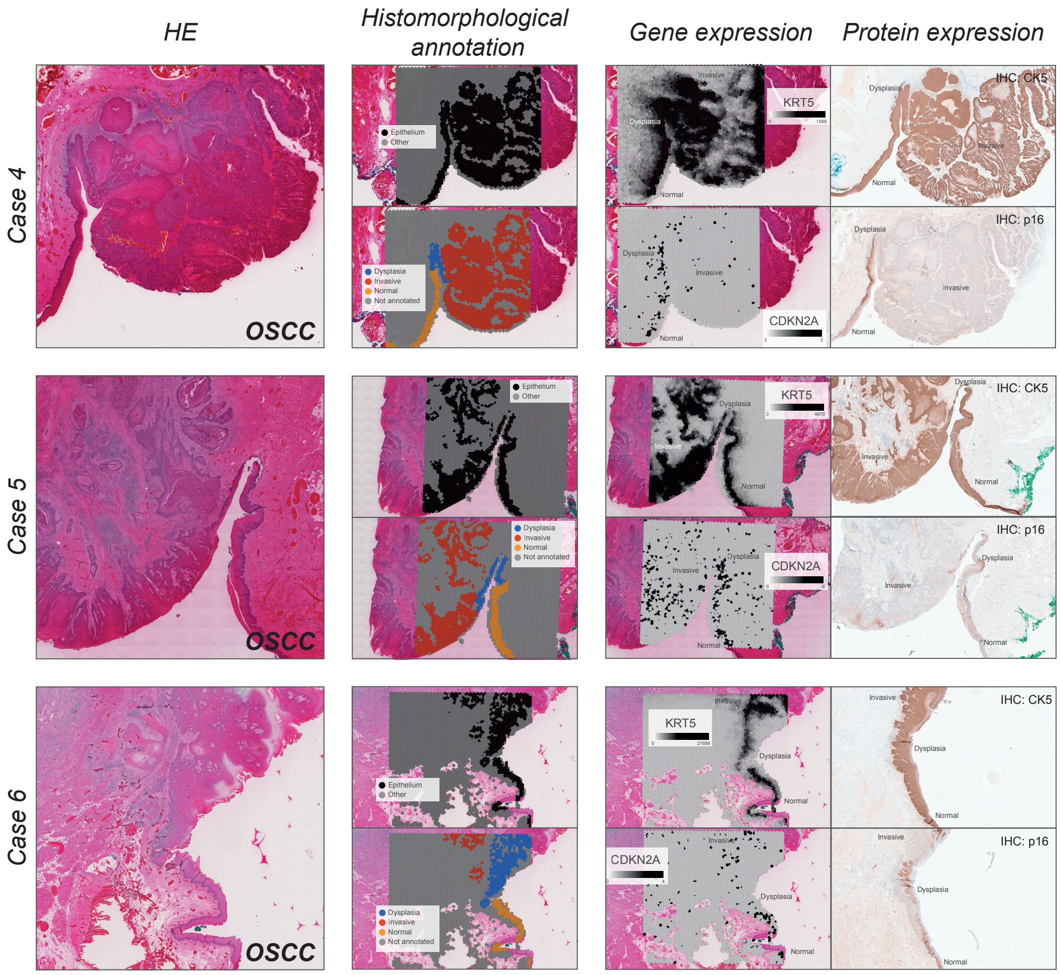

Figure S1. *The three included cases with HPV-independent oral squamous cell carcinoma (OSCC), presented by their histomorphology (HE), their manually annotations (Histomorphological annotation), their spatial gene expression (KRT5 and CDKN2A) and their protein expression (CK5 and p16) by immunohistochemistry.* The protein expression of CK5 was highly correlated visually to gene expression of KRT5 and histomorphological annotations (epithelium). No overexpression of p16 protein by IHC, nor any CDK2NA gene overexpression was observed. Therefore, we considered the gene-expression of the Visium assay, and our annotations validated.

Case 1

10x GENOMICS Space Ranger • count

P28354_101

Summary | Gene Expression

4,022
Number of Spots Under Tissue

49,096
Mean Reads per Spot

5,640
Median Genes per Spot

Sequencing

Number of Reads	197,463,938
Valid Barcodes	98.8%
Valid UMIs	100.0%
Sequencing Saturation	33.6%
Q30 Bases in Barcode	91.8%
Q30 Bases in Probe Read	91.7%
Q30 Bases in UMI	87.8%

Mapping

Reads Mapped to Probe Set	98.4%
Reads Mapped Confidently to Probe Set	98.1%
Reads Mapped Confidently to the Filtered Probe Set	95.1%

Image

Tissue Detection and Fiducial Alignment

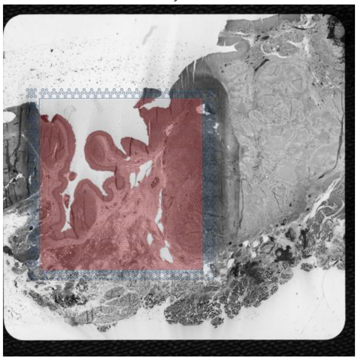

CytAssist Image Alignment

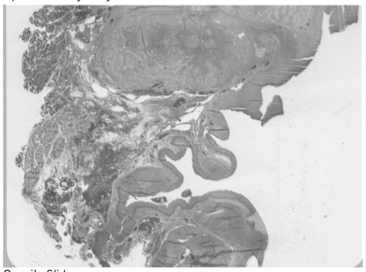

Opacity Slider

Spots

Fraction Reads in Spots Under Tissue	94.5%
Mean Reads per Spot	49,096
Mean Reads Under Tissue per Spot	45,880
Median UMI Counts per Spot	15,508
Median Genes per Spot	5,640
Genes Detected	18,072

Sample

Sample ID	P28354_101
Sample Description	
Chemistry	Visium V4 Slide - FFPE v2
Slide Serial Number	V43J12-299-A1
Reference Path	….0/reference/refdata-gex-GRCh38-2020-A
Probe Set Name	Visium Human Transcriptome Probe Set v2.0
Number of Genes Targeted	18085
Transcriptome	GRCh38-2020-A
Pipeline Version	spaceranger-2.0.1
Image Reorientation	On
Filter Probes	On

Case 2

10x GENOMICS Space Ranger • count

P30502_101-3B

Summary | Gene Expression

2,045
Number of Spots Under Tissue

89,934
Mean Reads per Spot

5,216
Median Genes per Spot

Sequencing

Number of Reads	183,914,138
Valid Barcodes	98.8%
Valid UMIs	100.0%
Sequencing Saturation	66.1%
Q30 Bases in Barcode	97.6%
Q30 Bases in Probe Read	97.5%
Q30 Bases in UMI	98.2%

Mapping

Reads Mapped to Probe Set	98.3%
Reads Mapped Confidently to Probe Set	97.5%
Reads Mapped Confidently to the Filtered Probe Set	93.5%

Image

Tissue Detection and Fiducial Alignment

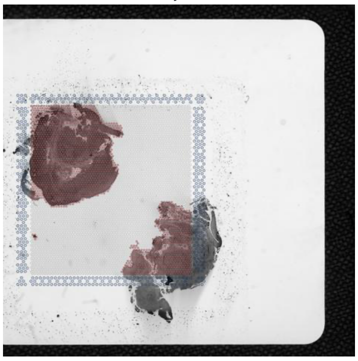

CytAssist Image Alignment

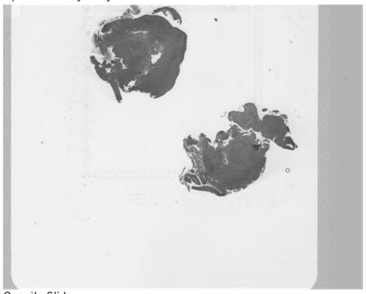

Opacity Slider

Spots

Fraction Reads in Spots Under Tissue	97.3%
Mean Reads per Spot	89,934
Mean Reads Under Tissue per Spot	86,475
Median UMI Counts per Spot	13,611
Median Genes per Spot	5,216
Genes Detected	18,059

Sample

Sample ID	P30502_101-3B
Sample Description	
Chemistry	Visium V4 Slide - FFPE v2
Slide Serial Number	V43T14-037-A1
Reference Path	…a/References/refdata-gex-GRCh38-2020-A
Probe Set Name	Visium Human Transcriptome Probe Set v2.0
Number of Genes Targeted	18085
Transcriptome	GRCh38-2020-A
Pipeline Version	spaceranger-2.1.1
Image Reorientation	On
Loupe Manual Alignment	P30502_101_V43T14-037-A1.json
Filter Probes	On

Case 3

10x GENOMICS Space Ranger • count

P30502_201-3C

Summary | Gene Expression

2,949
Number of Spots Under Tissue

46,302
Mean Reads per Spot

4,241
Median Genes per Spot

Sequencing

Number of Reads	136,545,934
Valid Barcodes	98.7%
Valid UMIs	100.0%
Sequencing Saturation	40.8%
Q30 Bases in Barcode	97.6%
Q30 Bases in Probe Read	97.6%
Q30 Bases in UMI	98.3%

Mapping

Reads Mapped to Probe Set	98.2%
Reads Mapped Confidently to Probe Set	96.6%
Reads Mapped Confidently to the Filtered Probe Set	91.2%

Image

Tissue Detection and Fiducial Alignment

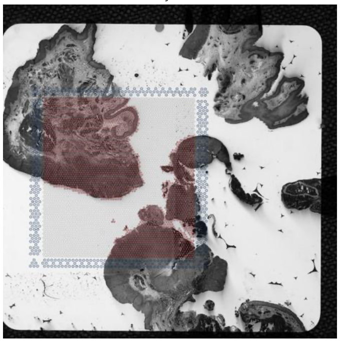

CytAssist Image Alignment

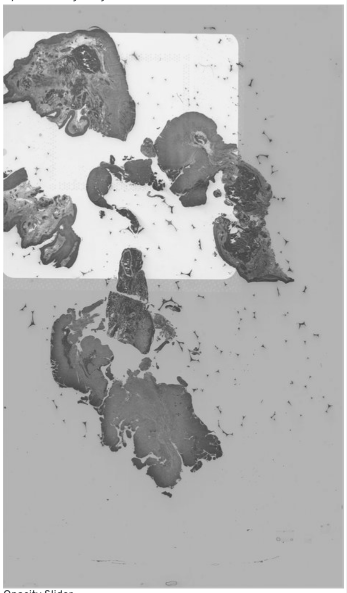

Opacity Slider

Spots

Fraction Reads in Spots Under Tissue	99.0%
Mean Reads per Spot	46,302
Mean Reads Under Tissue per Spot	45,212
Median UMI Counts per Spot	9,865
Median Genes per Spot	4,241
Genes Detected	18,064

Sample

Sample ID	P30502_201-3C
Sample Description	
Chemistry	Visium V4 Slide - FFPE v2
Slide Serial Number	V43T14-037-D1
Reference Path	…a/References/refdata-gex-GRCh38-2020-A
Probe Set Name	Visium Human Transcriptome Probe Set v2.0
Number of Genes Targeted	18085
Transcriptome	GRCh38-2020-A
Pipeline Version	spaceranger-2.1.1
Image Reorientation	On
Loupe Manual Alignment	P30502_201_V43T14-037-D1.json
Filter Probes	On

Case 4

10x GENOMICS Space Ranger • count

P29827_201-2E

Summary | Gene Expression

4,431
Number of Spots Under Tissue

36,229
Mean Reads per Spot

4,320
Median Genes per Spot

Sequencing

Number of Reads	160,529,054
Valid Barcodes	98.9%
Valid UMIs	100.0%
Sequencing Saturation	57.4%
Q30 Bases in Barcode	95.3%
Q30 Bases in Probe Read	94.3%
Q30 Bases in UMI	95.1%

Mapping

Reads Mapped to Probe Set	98.5%
Reads Mapped Confidently to Probe Set	97.0%
Reads Mapped Confidently to the Filtered Probe Set	94.2%

Image

Tissue Detection and Fiducial Alignment

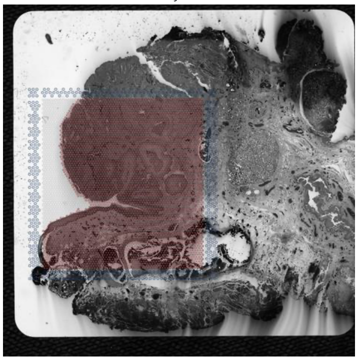

CytAssist Image Alignment

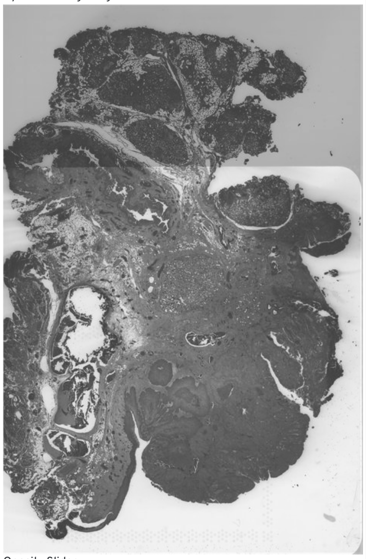

Opacity Slider

Spots

Fraction Reads in Spots Under Tissue	99.7%
Mean Reads per Spot	36,229
Mean Reads Under Tissue per Spot	35,704
Median UMI Counts per Spot	9,047
Median Genes per Spot	4,320
Genes Detected	18,069

Sample

Sample ID	P29827_201-2E
Sample Description	
Chemistry	Visium V4 Slide - FFPE v2
Slide Serial Number	V43A17-023-A1
Reference Path	…a/References/refdata-gex-GRCh38-2020-A
Probe Set Name	Visium Human Transcriptome Probe Set v2.0
Number of Genes Targeted	18085
Transcriptome	GRCh38-2020-A
Pipeline Version	spaceranger-2.1.0
Image Reorientation	On
Loupe Manual Alignment	P29827_201_V43A17-023-A1.json
Filter Probes	On

Case 5

10x GENOMICS Space Ranger • count

P29827_304-2M

Summary | Gene Expression

4,572
Number of Spots Under Tissue

39,203
Mean Reads per Spot

3,994
Median Genes per Spot

Sequencing

Number of Reads	179,236,937
Valid Barcodes	99.0%
Valid UMIs	100.0%
Sequencing Saturation	46.2%
Q30 Bases in Barcode	95.6%
Q30 Bases in Probe Read	94.3%
Q30 Bases in UMI	95.4%

Mapping

Reads Mapped to Probe Set	98.6%
Reads Mapped Confidently to Probe Set	97.7%
Reads Mapped Confidently to the Filtered Probe Set	95.3%

Image

Tissue Detection and Fiducial Alignment

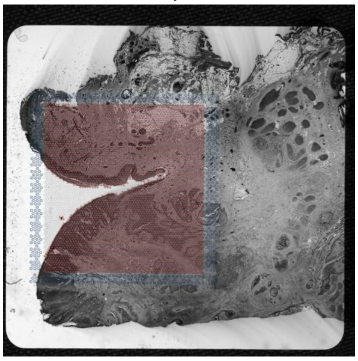

CytAssist Image Alignment

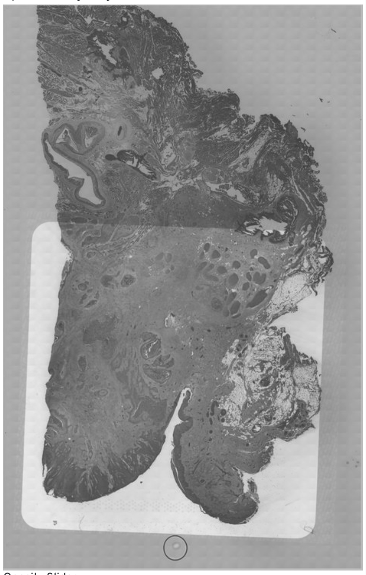

Opacity Slider

Spots

Fraction Reads in Spots Under Tissue	98.4%
Mean Reads per Spot	39,203
Mean Reads Under Tissue per Spot	38,187
Median UMI Counts per Spot	7,488
Median Genes per Spot	3,994
Genes Detected	18,070

Sample

Sample ID	P29827_304-2M
Sample Description	
Chemistry	Visium V4 Slide - FFPE v2
Slide Serial Number	V43A17-010-D1
Reference Path	…a/References/refdata-gex-GRCh38-2020-A
Probe Set Name	Visium Human Transcriptome Probe Set v2.0
Number of Genes Targeted	18085
Transcriptome	GRCh38-2020-A
Pipeline Version	spaceranger-2.1.0
Image Reorientation	On
Loupe Manual Alignment	P29827_304_V43A17-010-D1.json
Filter Probes	On

Case 6

10x GENOMICS Space Ranger • count

P28354_102

Summary | Gene Expression

3,482
Number of Spots Under Tissue

53,831
Mean Reads per Spot

2,528
Median Genes per Spot

Sequencing

Number of Reads	187,440,851
Valid Barcodes	98.7%
Valid UMIs	100.0%
Sequencing Saturation	67.0%
Q30 Bases in Barcode	91.6%
Q30 Bases in Probe Read	90.6%
Q30 Bases in UMI	87.6%

Mapping

Reads Mapped to Probe Set	97.8%
Reads Mapped Confidently to Probe Set	96.7%
Reads Mapped Confidently to the Filtered Probe Set	94.1%

Image

Tissue Detection and Fiducial Alignment

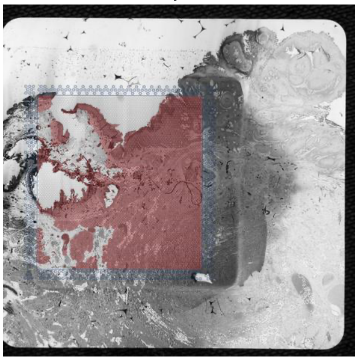

CytAssist Image Alignment

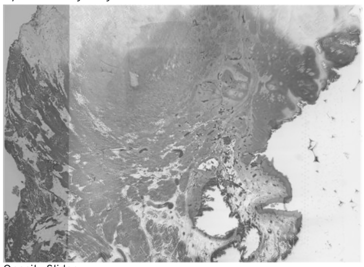

Opacity Slider

Spots

Fraction Reads in Spots Under Tissue	90.4%
Mean Reads per Spot	53,831
Mean Reads Under Tissue per Spot	48,079
Median UMI Counts per Spot	4,388
Median Genes per Spot	2,528
Genes Detected	18,057

Sample

Sample ID	P28354_102
Sample Description	
Chemistry	Visium V4 Slide - FFPE v2
Slide Serial Number	V43J12-299-D1
Reference Path	….0/reference/refdata-gex-GRCh38-2020-A
Probe Set Name	Visium Human Transcriptome Probe Set v2.0
Number of Genes Targeted	18085
Transcriptome	GRCh38-2020-A
Pipeline Version	spaceranger-2.0.1
Image Reorientation	On
Filter Probes	On

Case 7

10x GENOMICS Space Ranger • count

P30502_301-3D

Summary | Gene Expression

4,739
Number of Spots Under Tissue

20,821
Mean Reads per Spot

3,653
Median Genes per Spot

Sequencing

Number of Reads	98,671,419
Valid Barcodes	98.9%
Valid UMIs	100.0%
Sequencing Saturation	55.3%
Q30 Bases in Barcode	97.5%
Q30 Bases in Probe Read	97.7%
Q30 Bases in UMI	98.2%

Mapping

Reads Mapped to Probe Set	98.6%
Reads Mapped Confidently to Probe Set	98.0%
Reads Mapped Confidently to the Filtered Probe Set	95.3%

Image

Tissue Detection and Fiducial Alignment

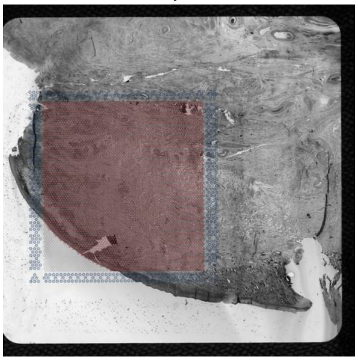

CytAssist Image Alignment

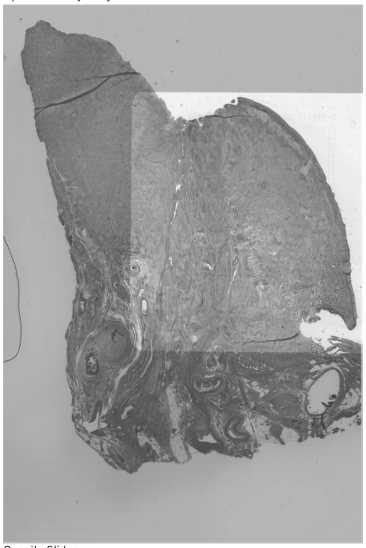

Opacity Slider

Spots

Fraction Reads in Spots Under Tissue	99.8%
Mean Reads per Spot	20,821
Mean Reads Under Tissue per Spot	20,554
Median UMI Counts per Spot	6,307
Median Genes per Spot	3,653
Genes Detected	18,055

Sample

Sample ID	P30502_301-3D
Sample Description	
Chemistry	Visium V4 Slide - FFPE v2
Slide Serial Number	V43T14-051-A1
Reference Path	…a/References/refdata-gex-GRCh38-2020-A
Probe Set Name	Visium Human Transcriptome Probe Set v2.0
Number of Genes Targeted	18085
Transcriptome	GRCh38-2020-A
Pipeline Version	spaceranger-2.1.1
Image Reorientation	On
Loupe Manual Alignment	P30502_301_V43T14-051-A1.json
Filter Probes	On

Case 8

10x GENOMICS Space Ranger • count

P30502_401-3E

Summary | Gene Expression

3,010
Number of Spots Under Tissue

37,602
Mean Reads per Spot

6,396
Median Genes per Spot

Sequencing

Number of Reads	113,181,548
Valid Barcodes	98.9%
Valid UMIs	100.0%
Sequencing Saturation	51.1%
Q30 Bases in Barcode	97.5%
Q30 Bases in Probe Read	97.7%
Q30 Bases in UMI	98.2%

Mapping

Reads Mapped to Probe Set	98.5%
Reads Mapped Confidently to Probe Set	98.2%
Reads Mapped Confidently to the Filtered Probe Set	95.5%

Image

Tissue Detection and Fiducial Alignment

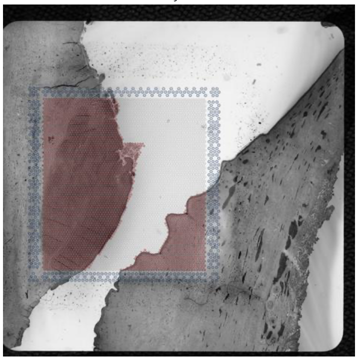

CytAssist Image Alignment

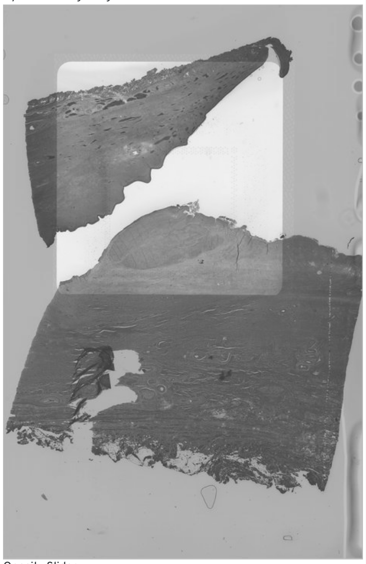

Opacity Slider

Spots

Fraction Reads in Spots Under Tissue	97.1%
Mean Reads per Spot	37,602
Mean Reads Under Tissue per Spot	36,090
Median UMI Counts per Spot	17,542
Median Genes per Spot	6,396
Genes Detected	18,050

Sample

Sample ID	P30502_401-3E
Sample Description	
Chemistry	Visium V4 Slide - FFPE v2
Slide Serial Number	V43T14-051-D1
Reference Path	…a/References/refdata-gex-GRCh38-2020-A
Probe Set Name	Visium Human Transcriptome Probe Set v2.0
Number of Genes Targeted	18085
Transcriptome	GRCh38-2020-A
Pipeline Version	spaceranger-2.1.1
Image Reorientation	On
Loupe Manual Alignment	P30502_401_V43T14-051-D1.json
Filter Probes	On
